# Chronic polyarthritis as the first manifestation of childhood systemic polyarteritis nodosa

**DOI:** 10.1590/S1679-45082017RC3783

**Published:** 2017

**Authors:** Glaucia Vanessa Novak, Koken Hayashi, Kohei Sampa, Yosuke Okumura, Gabriela Ribeiro Viola Ferreira, Clovis Artur Silva

**Affiliations:** 1 Instituto da Criança, Hospital das Clínicas, Faculdade de Medicina, Universidade de São Paulo, São Paulo, SP, Brazil.

**Keywords:** Polyarteritis nodosa/diagnosis, Arthritis, infectious, Streptococcal infections, Vasculitis, Case reports

## Abstract

Arthritis has been reported as an acute pattern, generally evanescent with oligoarthritis, mostly affecting knees and ankles in childhood systemic polyarteritis nodosa. However, chronic polyarthritis with morning stiffness mimicking juvenile idiopathic arthritis has not been reported. We describe the case of a 4-year old girl who had additive and chronic polyarthritis with edema, tenderness, pain on motion and morning stiffness for 2 months. After 45 days, she also presented painful subcutaneous nodules and erythematous-violaceous lesions in the extensor region of upper and lower limbs. She was admitted to university hospital due to high fever, malaise, myalgia, anorexia, loss of weight (1kg), painful skin lesions and severe functional disability. She was bedridden by chronic polyarthritis with limitation on motion. Systolic and diastolic blood pressures were greater than 95^th^ percentile for height. Urine protein/creatinine ratio was 0.39g/day, and immunological tests were negative. Anti-streptolysin O was 1,687UI/mL. Skin biopsy revealed necrotizing vasculitis in medium- and small-sized vessels compatible with polyarteritis nodosa. Therefore, we had the diagnosis of systemic polyarteritis nodosa. Prednisone 2mg/kg/day was administered with complete resolution of skin lesions and arthritis, and improvement of proteinuria (0.26g/day) after 15 days. The diagnosis of childhood systemic polyarteritis nodosa should be considered for patients with chronic polyarthritis associated to cutaneous vasculitis triggered by streptococcal infection.

## RESUMO

Na poliarterite nodosa sistêmica pediátrica, a artrite caracteriza-se pelo padrão agudo, geralmente evanescente, com oligoartrite, e afeta principalmente joelhos e tornozelos. No entanto, a poliartrite crônica com rigidez matinal e simulando artrite idiopática juvenil ainda não foi relatada. Descrevemos o caso de uma menina de 4 anos que apresentou poliartrite crônica aditiva com edema, dor à palpação e movimento, e rigidez matinal por 2 meses. Após 45 dias, também apresentou nódulos subcutâneos dolorosos e lesões eritêmato-violáceas na região extensora dos membros superiores e inferiores. Foi internada no hospital universitário por conta de febre alta, mal-estar, mialgia, anorexia, perda de peso (1kg), lesões de pele muito dolorosas e incapacidade funcional grave. Estava restrita ao leito devido à poliartrite crônica com limitação do movimento. Pressões sistólica e diastólica foram maiores que percentil 95 para altura. Relação proteína/creatinina urinária estava 0,39g/dia, e os testes imunológicos foram negativos. Antiestreptolisina O era 1.687UI/mL. A biópsia de pele revelou vasculite necrosante de vasos de pequeno e médio calibre, compatível com poliarterite nodosa. Portanto, foi realizado o diagnóstico de poliarterite nodosa sistêmica. Foi administrada prednisona 2mg/kg/dia com resolução completa das lesões de pele e da artrite, além de melhora da proteinúria (0,26g/dia) após 15 dias. O diagnóstico de poliarterite nodosa sistêmica pediátrica deve ser considerado em pacientes com poliartrite crônica associado a lesões cutâneas vasculíticas, sendo a infecção estreptocócica um importante fator desencadeante.

## INTRODUCTION

Childhood systemic polyarteritis nodosa (PAN) is a rare primary vasculitis characterized by necrotizing inflammatory abnormalities in small and/or medium-sized arteries.^1-8^ The European League Against Rheumatism (EULAR), the Paediatric Rheumatology International Trials Organisation (PRINTO) an the Paediatric Rheumatology European Society (PRES) defined new validated criteria for childhood PAN diagnosis. This new criteria had high specificity and excellent Kappa-agreement for diagnosis of this systemic vasculitis between the classification consensus panel and attending physicians.^2^

The main organs involved at the onset of childhood PAN are skin, muscles, bones and kidneys. The musculoskeletal features include arthralgia, myalgia and arthritis.^1,5^ This later manifestation has been described in 7.7% of cases at disease onset.^5^Arthritis has been reported as an acute pattern, generally evanescent with oligoarthritis, usually affecting knees and ankles.^8^ However, to our knowledge, chronic polyarthritis with morning stiffness mimicking juvenile idiopathic arthritis (JIA) has not been reported yet.

Therefore, from January 1983 to December 2014, 5,977 patients were followed-up at the Pediatric Rheumatology Unit of the *Instituto da Criança da Faculdade de Medicina da Universidade de São Paulo*, and 34 (0.56%) of them met the EULAR/PRINTO/PRES classification criteria for childhood PAN.^2^ One of them (2.9%) had childhood PAN with chronic polyarthritis mimicking JIA and is described in this case report.

## CASE REPORT

A 4-year old girl presented acute tonsillitis and was treated with benzathine penicillin. She also had additive and chronic polyarthritis with edema, tenderness and pain on motion during 2 months. The affected joints were elbows, right wrist, all proximal interphalangeal joints, knees, ankles and first metatarsophalangeal joint.

The patient presented morning stiffness during 30 to 60 minutes associated with limited motion in elbows, right wrist and knees, and was diagnosed as JIA prior to coming to our service.

**Figure 1:**
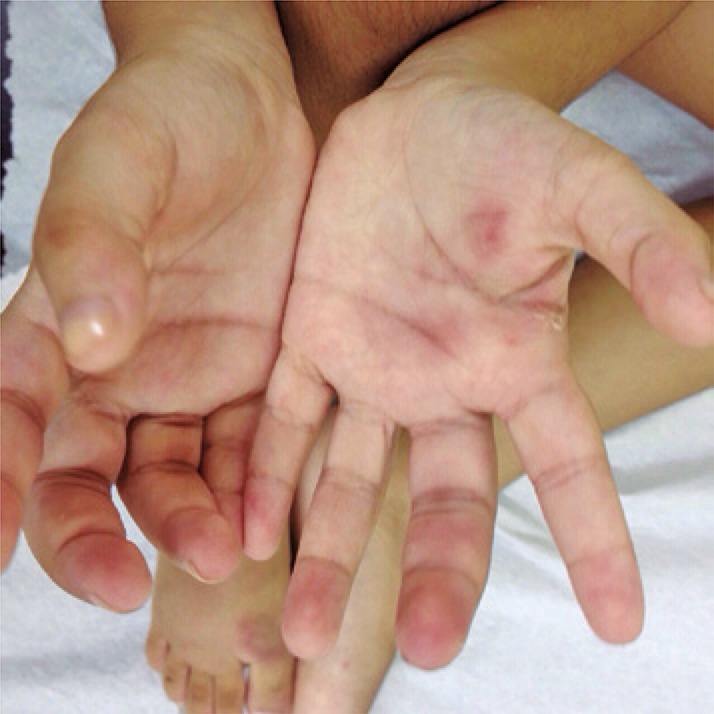
Painful subcutaneous nodules and erythematous-violaceous lesions

**Figure 2:**
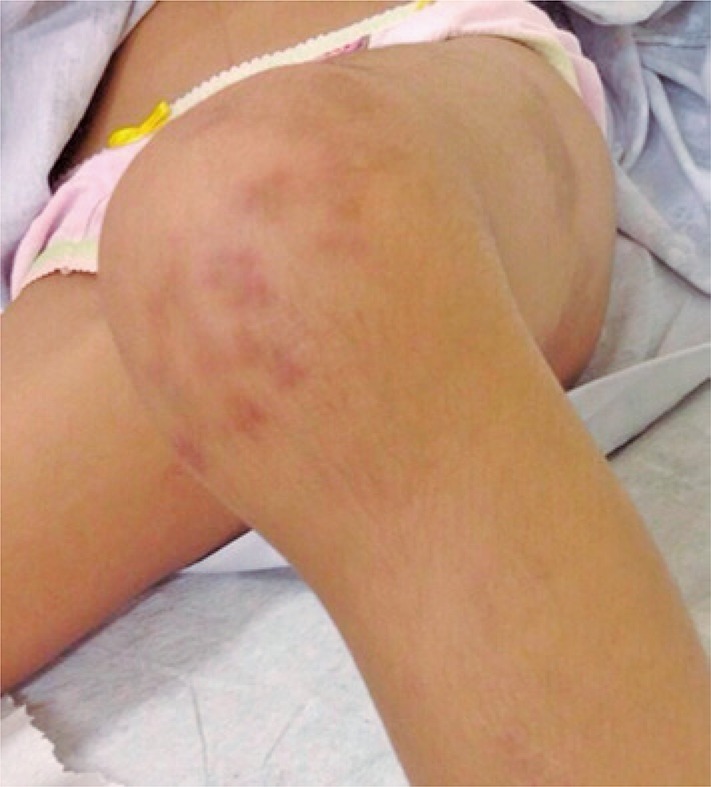
Arthritis on the knee and erythematous-violaceous lesions

**Figure 3:**
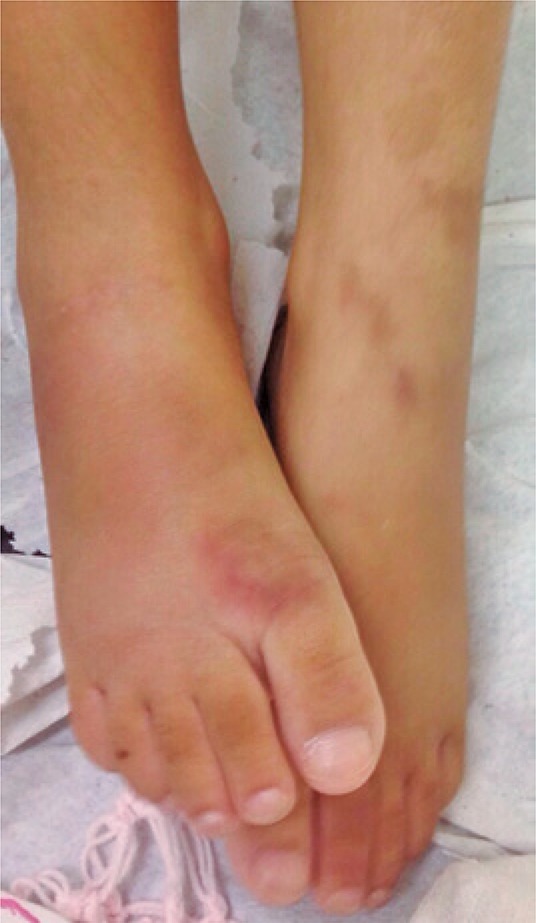
Erythematous lesions on the extensor region of the lower limbs

After 45 days, she also presented painful subcutaneous nodules and erythematous-violaceous lesions in the extensor region of upper and lower limbs (Figures 1 and 3). She was admitted to the teaching hospital due to high fever, malaise, myalgia, anorexia, loss of 1.0kg, severely painful skin lesions and severe functional disability. She was bedridden by chronic polyarthritis with limitation on motion (Figure 2).

Systolic and diastolic blood pressures were greater than the 95^th^ percentile for height. She received naproxen 20mg/kg/day, paracetamol and tramadol. Laboratory tests showed hemoglobin 10.9g/dL, white blood cell (WBC) count 25,380/mm^3^ (67% neutrophils, 19% lymphocytes, 13% monocytes and 1% eosinophils), platelets 683,000/mm^3^, erythrocyte sedimentation rate 53mm/first hour, C-reactive protein 246.4mg/dL (normal range: 0 to 0.3mg/dL), aspartate aminotransferase 15U/L (normal range: 15 to 40U/L), alanine aminotransferase 9U/L (normal range: 10 to 35U/L), urea 24mg/dL (normal range: 15 to 45mg/dL) and creatinine 0.35mg/dL (normal range: 0.6 to 0.9mg/dL). C3 was 174mg/dL (normal range: 90 to 180mg/dL) and C4 was 21.2mg/dL (normal range: 10 to 40mg/dL). Anti-streptolysin O was 1,687UI/mL. Urinalysis was normal and urine protein/creatinine ratio was 0.39g/day.

Immunological tests, such as antinuclear antibodies (ANA), anti-double-stranded DNA (anti-dsDNA), anti-Sm, anti-RNP, antineutrophil cytoplasmic antibodies (ANCA), anti-topoisomerase, anti-Ro/SSA, anti-La/SSB, and IgG and IgM anticardiolipin antibodies were negative. Serologies for hepatitis (A, B and C) virus, HIV, VDRL, Epstein-Barr virus, cytomegalovirus and toxoplasmosis were also negative. Echocardiogram and Doppler ultrasonography of renal artery were normal. Thoracic and abdominal magnetic resonance angiographies were also normal.

Skin biopsy revealed necrotizing vasculitis in medium- and small-sized vessels compatible with PAN. Therefore, childhood PAN was diagnosed according to the EULAR/PRINTO/PRES criteria.^2^ Prednisone 2.0mg/kg/day was administered with complete resolution of skin lesions and arthritis, and also improvement of proteinuria (0.26g/day) after 15 days.

## DISCUSSION

We reported this unique case of childhood PAN with chronic polyarthritis and morning stiffness, observed in a 31-year period at our tertiary teaching hospital.

Of note, JIA is the most important cause of chronic arthritis with morning stiffness and fever in children and adolescents, however it is an exclusion diagnosis. In addition, childhood systemic lupus erythematosus may also present chronic polyarthritis, as the first manifestation of disease in 2.6% of patients, mimicking JIA.^9^ In our patient, the painful subcutaneous nodules, erythematous-violaceous lesions, myalgia and proteinuria >0.3g/24 hours, as well as absence of autoantibodies, indicated primary vasculitis. The skin biopsy confirmed necrotizing vasculitis in medium- and small-sized vessels, thus suggesting childhood PAN.

The clinical manifestations of childhood PAN, including cutaneous, musculoskeletal and renal findings, were observed in 71 to 78%, 40 to 83% and 2 to 25%,^1,5^ respectively. Interestingly, arthritis upon onset of childhood PAN was described in 7.7% of patients. The most important sites of arthritis are ankles and knees,^8^ as evidenced in our childhood PAN patient.

In 2008, the EULAR/PRINTO/PRES proposed the criteria for childhood PAN diagnosis, and studied 150 patients with this condition worldwide, including some Brazilian patients.^2^ According to these criteria, our patient was classified as childhood PAN due to the presence of the mandatory criteria (histopathologic findings or angiographic abnormalities), plus any of the five following criteria: skin involvement, myalgia, renal involvement, arterial hypertension and peripheral neuropathy.

The etiopathogenesis of childhood PAN remains unclear. Polyarteritis nodosa and cutaneous polyarteritis nodosa may be associated with acute serological or microbiological evidence of streptococcal infection, as observed in this case.^8^ Recurrence of this disease may also be triggered by this infection.^8^ The use of prophylaxis with benzathine penicillin, which is recommended for rheumatic fever, should also be indicated for patients who present recurrence of childhood PAN.^8^

The treatment of childhood PAN requires administration of high doses of corticosteroid, as prescribed to our patient. Immunosuppressive drugs, especially cyclophosphamide and azathioprine, are indicated in nonresponsive cases.^1,5^

## CONCLUSION

We described a rare case of childhood systemic polyarteritis nodosa that presented chronic polyarthritis as the initial manifestation of disease. We suggested that the diagnosis of childhood systemic polyarteritis nodosa should also be considered for patients with chronic polyarthritis and cutaneous lesions triggered by streptococcal infection.
